# Precision Cardio-oncology: Update on Omics-Based Diagnostic Methods

**DOI:** 10.1007/s11864-024-01203-6

**Published:** 2024-04-27

**Authors:** Ziyu Kuang, Miao Kong, Ningzhe Yan, Xinyi Ma, Min Wu, Jie Li

**Affiliations:** 1 Oncology Department, Guang’anmen Hospital, China Academy of Chinese Medical Sciences, Beijing, China; 2 Graduate School, Beijing University of Chinese Medicine, Beijing, China; 3 Cardiovascular Department, Guang’anmen Hospital, China Academy of Chinese Medical Sciences, Beijing, China

**Keywords:** Cardio-oncology, Cardiotoxicity, Omics, Precision medicine

## Abstract

Cardio-oncology is an emerging interdisciplinary field dedicated to the early detection and treatment of adverse cardiovascular events associated with anticancer treatment, and current clinical management of anticancer-treatment-related cardiovascular toxicity (CTR-CVT) remains limited by a lack of detailed phenotypic data. However, the promise of diagnosing CTR-CVT using deep phenotyping has emerged with the development of precision medicine, particularly the use of omics-based methodologies to discover sensitive biomarkers of the disease. In the future, combining information produced by a variety of omics methodologies could expand the clinical practice of cardio-oncology. In this review, we demonstrate how omics approaches can improve our comprehension of CTR-CVT deep phenotyping, discuss the positive and negative aspects of available omics approaches for CTR-CVT diagnosis, and outline how to integrate multiple sets of omics data into individualized monitoring and treatment. This will offer a reliable technical route for lowering cardiovascular morbidity and mortality in cancer patients and survivors.

## Introduction

Since the 1990s, the incidence of cancer has risen continuously [1]. Currently, radiotherapy, chemotherapy, molecular targeted inhibitors, targeted immune checkpoint inhibitors (ICIs), and other methods are used to treat advanced cancer [2]. However, most anti-tumor therapies result in varying degrees of cardiotoxicity, some are reversible and some are irreversible. For example, anthracyclines (e.g., doxorubicin, epirubicin) were the first chemotherapeutic agents to be reported to cause cardiotoxicity, and their adverse cardiovascular events were insidious and irreversible. With the continuous updating of clinical guidelines, molecular targeted inhibitors (e.g., HER2 inhibitor, tyrosine kinase inhibitor) and immunotherapy drugs (e.g., ICIs) are becoming more and more widely used in clinical practice, but the associated cardiovascular events are also constantly being reported. Compared with anthracycline-induced cardiovascular events, most of the cardiovascular events caused by molecular targeted inhibitors are nonspecific and reversible, which lead clinicians to often ignore the cardiovascular adverse events they cause [3]. The incidence of cardiovascular events caused by ICIs is low but relatively severe, and the mechanism of occurrence is not fully elucidated [4]. Cancer treatment–related cardiovascular toxicity (CTR-CVT) has become a common issue for both cardiologists and oncologists [5]. This resulted in the awareness of cardio-toxic effect of anticancer drugs and emergence of a new discipline: cardio-oncology.

To prevent CTR-CVT, it is crucial to develop a model for early diagnosis; however, no one biomarker can provide an accurate prognosis. Deep phenotypic profiles of CTR-CVT have been sought by researchers for the past 10 years, and improvements in multi-omics approaches have identified new information-rich biomarkers as well as biosignatures that show promise for use in clinical settings. In this review, we update current omics-based approaches to CTR-CVT diagnosis and attempt to propose a paradigm for developing a multi-omics combination (e.g., genomics, proteomics, or metabolomics) for optimal diagnosis to manage the early detection of CTR-CVT and further explore how to shape the future of personalized cardio-oncology.

## What is CTR-CVT?

Due to inconsistencies in cardiotoxicity endpoint markers, CTR-CVT is challenging to diagnose in clinical practice. Table 1 compares the definitions of CTR-CVT.

**Table 1:** Different clinical guidelines define CTR-CVT

Organization	Year	Guidelines	Definition	Ref
Chinese Society of Clinical Oncology	2023	Guidelines of Chinese society of clinical oncology (CSCO) Cardiovascular toxicity associated with cancer treatment	There are nine types including myocardial dysfunction and heart failure, coronary heart disease, valvular disease, arrhythmia, hypertension, thromboembolic disease, peripheral vascular disease and stroke, pulmonary hypertension, and pericardial disease	[6]
International Cardio-Oncology Society	2022	Defining cardiovascular toxicities of cancer therapies: an International Cardio-Oncology Society (IC-OS) consensus statement	There are five types of cardiac dysfunction (cardiomyopathy/heart failure), myocarditis, vasotoxicity, hypertension, and arrhythmias and QTc prolongation	[7•]
European Society of Cardiology(ESC)	2022	2022 ESC Guidelines on cardio-oncology developed in collaboration with the European Hematology Association (EHA), the European Society for Therapeutic Radiology and Oncology (ESTRO) and the International Cardio-Oncology Society (IC-OS)	Including cardiac dysfunction associated with cancer treatment, cardiomyopathy, myocarditis, hypertension, arrhythmia, etc., the CTR-CVT definition of central package disease and valvular heart disease (VHD) is the same as the definition of the general cardiology population	[8••]
American Heart Association	2021	Recognition, prevention, and management of arrhythmias and autonomic disorders in cardio-oncology: a scientific statement from the American Heart Association (AHA)	Including cardiac dysfunction associated with cancer treatment, cardiomyopathy, myocarditis, hypertension, arrhythmia, etc., the CTR-CVT definition of central package disease and VHD is the same as the definition of the general cardiology population	[9]

The most frequently documented cardiotoxicity caused by conventional chemotherapy medicines is anthracycline-induced cardiotoxicity (AIC) [10], which has undergone substantial research and is dose-dependent and irreversible. Doxorubicin (DOX)-mediated cardiotoxicity (DIC) has been shown to reduce survival and worsen prognoses [11], and DOX ≥ 400 mg/m^2^ is a high-risk factor for cardiotoxicity [12]. Anthracyclines are topoisomerase (topo) inhibitors that target topo IIα of cancer cells to prevent DNA replication and inhibit cell division; they also target topo IIβ expressed by cardiomyocytes to induce DNA double-strand breaks. These DNA double-strand breaks can also lead to abnormal mitochondrial structure [13] and oxidative stress [14] in cardiomyocytes, which results in progressive remodeling of the heart, and eventually results in heart failure. AIC is the most studied cardiotoxicity in the traditional sense of cardiotoxicity, but with the advent of molecular targeted drugs and ICIs, the pathogenesis of CTR-CVT has become more complex, with a variety of mechanisms that are overlapping, additive, or synergistic [15].

Targeted therapies like HER2 and tyrosine kinase inhibitors induced CTR-CVT which with symptoms and signs resembling those of patients with AIC, such as tachycardia, palpitations, and dyspnea with exertion, most reported cardiac effects are mild to moderate, nonspecific, and cause reversible cardiotoxicity [16]. Although its incidence is low, congestive heart failure may progress from these effects. Only 4% of patients receiving trastuzumab experience cardiac dysfunction; however, when it is paired with anthracyclines and cyclophosphamide, this number rises to 27% [17, 18]. Previous anthracycline exposure is thought to be one of the risk factors for trastuzumab-induced cardiotoxicity [19].

ICI is a monoclonal antibody antagonist that increases the body’s immune response to cancer cells by blocking intrinsic immune response downregulators, such as CTLA-4 and programmed cell death 1 (PD-1) or its ligand programmed cell death ligand 1 (PD-L1). Cardiotoxicity associated with ICI includes myocarditis, pericarditis, atherosclerosis, arrhythmias, and vasculitis [20]. ICI-associated myocarditis is the most common and possibly fatal adverse event, with a mortality rate approaching 50% [21]. In CAR-T cell therapy, autologous T cells are genetically engineered to express receptors that specifically target cancer cells. In addition to being an antitumor therapy, they produce a specific immunological response called cytokine release syndrome (CRS) [22]. High-grade CRS causes serious systemic abnormalities, including cardiovascular ones, such as arrhythmias, hemodynamic impairment, and cardiomyopathy.

Reducing CTR-CVT while aiming to offer optimum antitumor efficacy requires delicate balancing. However, it is interesting that many treatment medicines or methods only advocate symptom-based monitoring, whereas very few advocate routine baseline left ventricular ejection fraction (LVEF) evaluation and/or cardiac function monitoring [23]. In addition, the multi-targeted nature of many cancer therapies creates the potential for other untested cardiotoxicity; in addition, there are significant individual variations in cardiovascular damage and cancer responses, making it challenging to assess risks and benefits.

## Diagnosis of CTR-CVT based on omics techniques

**Fig. 1:**
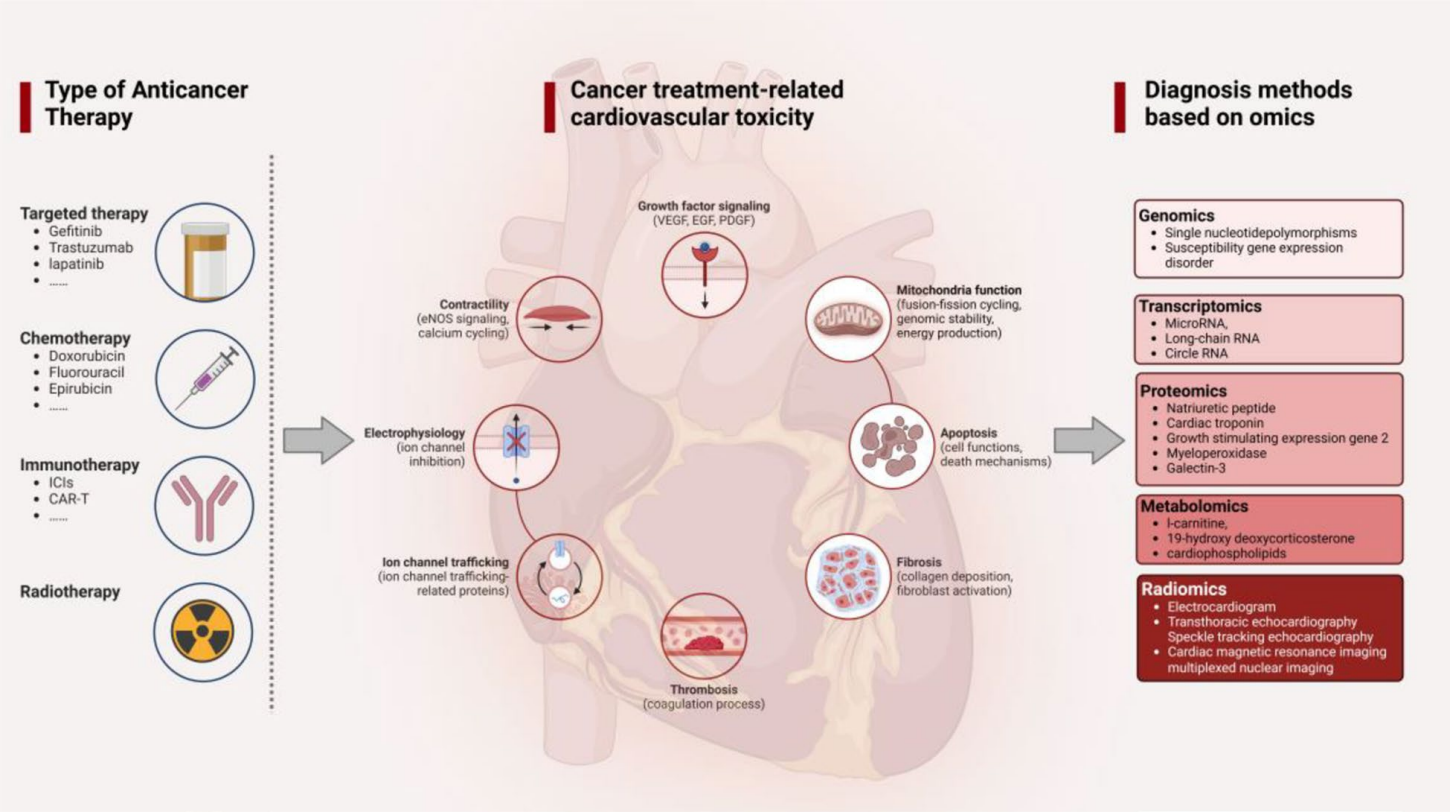
Omics classification for the diagnosis of CTR-CVD (Created with BioRender.com).

Radiomics is the most common omics used in CTR-CVT diagnosis, while genomics, transcriptomics, proteomics, and metabolomics can provide data related to gene expression, mRNA transcription, protein translation, protein modification, metabolites, and imaging changes during drug exposure [24] (Fig. 1).

### Genomics

Many factors affect the extent of cardiovascular damage from anticancer drug exposure. Although difficult to define, it is clear that individual factors contribute to differences in cardiotoxicity risk [25, 26], which explains why some patients develop cardiotoxicity during anticancer therapy while others tolerate high-dose chemotherapy. Therefore, genetic screening is beneficial in identifying patients susceptible to CTR-CVT, thereby achieving an early warning for CTR-CVT [27].

Single-nucleotide polymorphisms (SNPs) are important causes of human genetic variation as well as more complex structural variations [28]; with the development of human-induced pluripotent stem cell-derived cardiomyocytes (hiPSC-CMs), the potential risks and benefits of SNPs may be confirmed in cell models [29, 30]. For example, SNPs (rs1114049) of *solute carrier family 28 member 3* have been found to be protective against DIC in hiPSC-CMs [31], and an SNP (rs2229774) in *retinoic acid receptor γ* (*RARG*) is associated with an increased risk of AIC [32]. There is also a link between SNPs and DIC in ATP-binding cassette (ABC) transporters [33]. *Transient receptor potential 6* (*TRPC6*) gene alterations are putative risks for chemotherapy-induced congestive heart failure, and TRPC6 N338S is a gain-of-function mutant that may lead to DIC by increasing Ca^2+^ influx within cardiomyocytes [34•]. Trastuzumab-induced cardiotoxicity has been linked to two SNPs in the HER2/ERBB2 gene, rs1058808 and rs1136201 [35].

In addition to genetic alterations, epigenetic alterations are common in all forms of cancer, and abnormal DNA methylation may eventually lead to transcriptional gene silencing. DOX can lead to the downregulation of DNA methyltransferase 1 (DNMT1) enzyme activity, resulting in a decrease in DNA methylation [36]. For example, in one study, significantly differentially methylated regions were found in *SLFN12*, *IRF6*, and *RNF39* genes and promoters in patients with LVEF abnormalities after DOX treatment [37].

Some gene expression upregulation or knockdown also affects CTR-CVT susceptibility; for example, knocking down the expression of *activating transcription factor 4* (*ATF4*) exacerbates sorafenib-induced cardiomyocyte ferroptosis, and *ATF4* overexpression promotes cardiomyocyte survival [38]. In addition, anthracyclines are topo inhibitors; therefore, patients with high *topo II β* gene expression are often prone to cardiotoxicity [39], and inhibition of *topo II β* expression in cardiomyocytes would be a primary prevention strategy. However, the cost of genetic testing is higher than testing for traditional biomarkers, and genomics related to cardio-oncology is still in the experimental stage.

### Transcriptomics

Current applications of transcriptomics in CTR-CVT are focused on non-coding RNAs (ncRNAs), which are important regulatory factors in the cardiovascular system, even though they do not encode proteins [40].

MiRNA is an ncRNA about 22 nucleotides long that binds to an mRNA target sequence and inhibits translation or degradation of the target mRNA [41, 42]. MiRNA controls the basic functions of almost all cell types associated with the cardiovascular system, especially endothelial cells and cardiomyocytes [43, 44]. MiRNAs such as miR-1, miR-21, miR-34, miR-133, miR-208, and miR-499 are myocardial-enriched miRNAs, especially miR-1 and miR-133, which are also known as myocardial-specific miRNAs. They can be used as CTR-CVT-related biomarkers, and their sensitivity exceeds even that of traditional cardiac biomarkers, such as cardiac troponin (cTn) or natriuretic peptides (NPs) [45••, 46], and it can still be detected under extreme physical conditions (e.g., repeated freeze–thaw cycles, high temperature, pH changes, and long-term storage).

LncRNA is a less explored transcriptome in the cardiovascular system, but some lncRNAs play important roles in pathological processes of the cardiovascular system. LncRNA MIAT [47], lncRNA-NEAT1 [48], lncRNA NORAD [49], and lncRNA HOXB-AS3 [50] protect against AIC, but increased lncRNA PVT1 is positively correlated with cardiomyocyte damage caused by antitumor therapy [51]. In addition, the expression of lncRNA CMDL-1 was significantly downregulated in the DIC model [52]. However, compared with miRNA, lncRNA is relatively conserved in terms of nucleotide sequences, which somewhat limits its diversity.

CircRNA is a circular, single-stranded ncRNA and therefore exhibits greater structural stability than stranded ncRNA [53]. In cardiotoxicity, circ-INSR regulates apoptosis and metabolic pathways in cardiomyocytes and is downregulated during DIC and cardiac remodeling [54]. Circ-FOXO3 expression is significantly upregulated during radiotherapy-induced cardiotoxicity (*p* < 0.0001); in addition, circRNA functions as a sponge for miRNAs to influence cardiotoxicity. For example, circ-LTBP1 and circ-SKA3 can induce DIC [55, 56], and circ-0001312 inhibits DIC by silencing miR-409-3p [57]. Like lncRNA, circRNA has limited applicability in the field of cardio-oncology because it has not been sufficiently explored.

The discovery of ncRNA has enriched the knowledge of the mechanism of CTR-CVT to some extent; however, the techniques used to track these ncRNAs and their functions are often more complex than those for tracking proteins, potentially increasing costs. These new technologies will also require further validation before evidence-based guidelines can be established, and the balance between cardiac risk and anticancer efficacy must be optimized to provide maximum benefits. To date, the research has mainly been conducted as cell experiments and small-scale clinical research studies; however, based on current research results, its clinical prospects are broad.

### Proteomics

The value of deep phenotyping has been demonstrated in the study of CVD [58]. The proteome is a dynamic reflection of genes and their environment, and some specific proteins are released into the circulatory system when cardiomyocytes or vascular endothelial cells are damaged [55]. NPs and cTn are two recognized biomarkers of cardiomyocyte damage, which was recommended by the 2022 ESC Guidelines because they are less invasive and easily repeated [8••]. After antineoplastic therapy, approximately 22% of patients have cTn or BNP and N-terminal pre-B NP (NT-proBNP) above the cutoff value [59].

cTn is highly sensitive to myocardial injury and is commonly used in the diagnosis and risk stratification of acute coronary syndrome (ACS). Among these, cTnI has strong specificity and is only expressed in the myocardium, while cTnT is mainly expressed in the myocardium and expressed at low levels in skeletal muscle. The long diagnostic window is one of the obvious advantages of cTn as a biomarker; however, the biological half-life of cTn is relatively short. Thus, cTn levels may depend on the timing of sampling, and if there is no sustained damage, the serum concentration of cTn may quickly return to baseline levels [60]. Questions remain about whether cTnI or cTnT should be preferred for cardiotoxicity evaluation. The highly sensitive cTn (hs-cTn) is effective for monitoring adverse cardiac events associated with chemoradiotherapy in a variety of malignancies [61], and a meta-analysis showed that the area under the curve for diagnosis of CTR-CVT with elevated hs-cTnT increased from 0.83 to 0.90 (95% CI, 0.87–0.92) at 3 to 6 months, suggesting that hs-cTnT is more valuable for early diagnosis of CTR-CVT than echocardiography [62]. Compared with conventional cTn, the lower detection limit of hs-cTnT is very low at 0.003 ng/mL [63]. Although cTn may be a common indicator of cardiomyocyte injury, the predicted threshold remains undetermined, and simply using the ACS threshold does not provide a good indication of myocardial damage.

BNP and NT-proBNP are quantitative markers of heart failure, and when ventricular volume or pressure rises, cardiomyocytes secrete large amounts of BNP or NT-proBNP into the circulatory system. Serum BNP levels after the last round of anthracycline chemotherapy are independent predictors of cardiotoxicity (*p* = 0.047), with an optimal diagnostic threshold of 107.9 ng/L [64], and high NT-proBNP levels (OR 22.0, 95% CI 5.7–85.4, *p* < 0.0001) are positively associated with trastuzumab-induced cardiotoxicity [65]. While the mechanism of transient BNP decline following antitumor drug administration is currently unknown, these results may help assess myocardial damage caused by cancer drugs. The regulation of NPs is more complex than that of cTn; e.g., a transient increase in NT-proBNP occurs with exposure to anthracyclines when LVEF is relatively stable, suggesting a degree of reversibility or threshold effects in cardiac homeostatic repair mechanisms [66].

Growth-stimulating expression gene 2 (ST2) is divided into two subtypes: a transmembrane ligand ST2 (ST2L) and soluble ST2 (sST2) [67]. sST2 can bind to IL-33 and inhibit IL-33/ST2L signal transduction [68]; therefore, too much sST2 may result in insufficient protection for the myocardium when damaged, and ST2L is beneficial but sST2 is harmful to the body. Cardiac hypertrophy, fibrosis, and ventricular dysfunction are associated with abnormal sST2 levels [69]. One 3-year follow-ups in radiotherapy patients showed that elevated sST2 levels were positively associated with systolic function and LVEF deterioration [70], and another study showed a 1.6-fold increase in sST2 from baseline in patients taking epirubicin, cyclophosphamide, trastuzumab, or lapatinib (*p* < 0.001) [71]. However, ST2 is not tissue-specific [72, 73]; thus, CTR-CVT cannot be evaluated as a single index at the time of diagnosis, and joint diagnoses must be carried out in combination with other relevant biological indicators.

Myeloperoxidase (MPO) is an enzyme secreted by myeloid cells and is key in mediating cardiotoxicity caused by DOX [74]. One meta-analysis showed that an increased risk of CTR-CVT was associated with early pre- and post-MPO assessment (HR 1.16, 90% CI 1.02–1.32) [75]; however, in addition to cardiac injury, the tumor itself may be responsible for elevated MPO levels after antineoplastic therapy, which partly contributes to the imprecision of MPO results. Thus, the accuracy of MPO requires additional verification.

Galectin-3 (Gal-3), a potential biomarker for predicting early- or late-onset cardiotoxicity, is a highly conserved β-galactoprotein of around 30 kDa [76]. High levels of Gal-3 are positively associated with a risk of death in patients with heart failure [77], and Gal-3 is an advantageous marker because it is detectable in saliva [51]. However, clinically, Gal-3 may be affected by the pathophysiological overlap between CVD and cancer [78••]. Therefore, more multicenter, large sample size studies are needed to confirm the predictive potential of Gal-3 for CTR-CVT.

### Metabolomics

Metabolomics is a novel high-throughput bioanalytical technology for identifying and quantifying small-molecule compounds (molecular weight < 1500 Da) found in biological systems [79], and differences in individual genomes, transcriptomes, and proteomes are reflected in metabolomes. Metabolomics is one of the most effective methods for detecting early changes in cellular responses to harmful injury [80]. Targeted and non-targeted metabolomics has been used to identify circulating metabolites associated with drug-induced cardiotoxicity [81]. By analyzing endogenous and exogenous small molecules that act as substrates and products of metabolic processes, scientists have found that metabolomics can provide more information about subtle changes occurring in various biological processes and diseases. The use of analytical tools, such as nuclear magnetic resonance, mass spectrometry, and ultra-performance liquid chromatography (UPLC) has improved our understanding of the metabolome. There are multiple metabolic disorders between CVD and cancer [82]; similarly, changes in metabolite profiles over time, such as after medication administration, can be used to determine an individual’s response to treatment and guide subsequent disease management. Cardiometabolic alterations may play key roles in the development of AIC, and Li et al. [83] have shown that highly specific biomarkers in rat cardiotoxicity models include l-carnitine and 19-hydroxy deoxycorticosterone and used predictive models combined with these metabolites to predict cardiotoxicity with a prediction rate of up to 90%. In vivo experiments in rats showed that DOX has a sex-specific effect on cardio-phospholipids, particularly cardiolipin [84]. However, the application of metabolomics to cardiotoxicity is in its infancy, and it is easily disturbed by many internal and external potential factors, such as environmental temperature changes, body fluid pH, and drug intervention [10]. There is also a lack of reliable evidence about how to apply metabolites with significant differences in animal experiments to clinical studies.

### Radiomics

Cardiovascular imaging plays an important role in identifying patients with subclinical CVD, determining the extent of pre-existing cardiac comorbidities, and as a reference to identify changes during treatment and long-term follow-up. Current guidelines recommend several strategies for screening and testing cardiotoxicity, including electrocardiography, echocardiography, nuclear imaging, and cardiac magnetic resonance imaging (CMR) [8••].

Due to its low cost and non-invasive nature, an electrocardiogram (ECG) is the easiest test for evaluating CTR-CVT. In a retrospective study, T-wave changes and QT interval prolongation were shown to be early indicators of CTR-CVT in patients with DIC [85], and ECG was recommended as class IC evidence in the baseline cardiotoxicity risk assessment. ECGs are recommended for all patients starting cancer therapy as part of a baseline cardiovascular risk assessment [8••]. However, there are many confounding factors for ECGs, and their accuracy is questionable because asymptomatic CTR-CVT often cannot be directly observed by ECG changes.

Transthoracic echocardiography (TTE) is the most versatile, accessible, and commonly used method for evaluating patients undergoing chemotherapy, and TTE is used in screening for drug baselines for almost all potential cardiotoxicity. Two-dimensional echocardiography (2DE) is currently one of the most used clinical methods to detect CTR-CVT. LVEF is the most widely used parameter to evaluate cardiotoxicity because one of the main toxicities resulting from anthracycline and trastuzumab use is LVEF decline. In one study, the degree of decline in 2DE-LVEF was the only independent predictor of trastuzumab-induced cardiotoxicity [86], but the method calculated left ventricular volume and LVEF using geometric assumptions and clear intimal boundaries, which have led to apical fluoroscopic reduction, resulting in widely variable measurements [87]. Compared with 2DE, 3DE can detect small LVEF changes, with a test variability of 5.6% compared with 9.8% for 2DE [88], but it is limited by operator expertise, standardized methods, the current frame rate, and the temporal and spatial resolution of 3DE imaging [89].

Cardiac MRI (CMR) can identify small changes in left ventricular volume and ejection fraction during treatment [90], and CMR combined with advanced gadolinium contrast enhancement can detect subtle irreversible damage to clear myocardial injury [91••]. Because early changes in LVEF due to cardiotoxicity are small, CMR is the best imaging alternative when echocardiography is not diagnostic. However, it is an expensive test and not compatible with metal implants.

Speckle tracking echocardiography (STE) quantitatively evaluates the deformation of the myocardium along the longitudinal, radial, and circumferential axes independently of angle, with better spatial resolution and insensitivity to signal noise [92]. Its common parameters are longitudinal strain, radial strain, circumferential strain, area strain, rotation angle, and its corresponding strain rate. Strain and strain rate reflect the ability and speed of the myocardium to deform under tension, respectively. Global longitudinal strain (GLS) can monitor the effects of antineoplastic drugs on the myocardium more sensitively and specifically than LVEF, and cardiotoxicity can be detected before LVEF drops significantly [93].

Multiplexed nuclear imaging (MUGA) may be used as a third-line diagnostic technique if LVEF cannot be assessed by TTE or CMR [8••]. MUGA scans ought to be avoided unless absolutely necessary because of radiation exposure and a lack of access to other crucial information, such as GLS [8••].

ESC guidelines recommend baseline LVEF and GLS for all patients evaluated for TTE prior to initiation of cancer treatment to stratify CVD risk and identify significant changes during treatment [8••]. However, it is worth noting that the main limitation of this approach is its low sensitivity for detecting cardiotoxicity in the early stages because left ventricular systolic function does not change significantly until borderline myocardial injury has occurred. Also, evidence of normal LVEF does not exclude the possibility of subsequent cardiac deterioration; therefore, it can be used as an indicator of subclinical dysfunction but is not a suitable prognostic indicator of disease [8••].

## Diagnosis using combined multi-omics approaches in CTR-CVT

Based on personalized medicine, using precision medicine is strategic to mitigate the adverse effects of drug toxicity in the treatment of CTR-CVT. This approach involves the integration of a combination of multi-omics techniques to identify or predict potential risks, along with comprehensive data analysis methods (Table 2) [94, 95]. Through this analysis, it was determined that existing clinical techniques for detecting CTR-CVT possess their own strengths and weaknesses. Therefore, the implementation of multi-omics approaches can offer the benefits of multiple diagnostic and treatment modalities. This can ultimately lead to precise prediction and diagnosis of CTR-CVT, making it a highly promising method.

**Table 2:** Multi-omics joint diagnosis mode of precision medicine

Multiomics combination	Specific indicators	Monitoring time point	Ref
Proteomics combined with echocardiography	hs-cTnI, GLS	3 months	[97]
	sST2, GLS, LVEF	NA	[70]
	hs-cTnI, MPO, LVEF	3 months	[98]
	MPO, cTnI	3 months	[99]
	cTnT, NT-proBNP, LV end-diastolic posterior wall thickness, LV thickness-to-dimension ratio	3 months	[100]
	cTn, GLS	NA	[101]
	The relative reduction of LV-GLS, the relative elevation of NT-proBNP	6 weeks after the first chemotherapy dose	[103]
Metabolomics combined with proteomics and echocardiography	citric acid, aconitic acid, LVEF	3 months	[107]
CMR combined with proteomics, genomics and echocardiography	CMR MAPSE, LVEF, miRNA-5-221p, miRNA-3-2p	Completion of chemotherapy and 6-month follow-up	[109]
	fast-SENC CMR, LVEF, cTnI, BNP	After 5 ± 2 months	[111••]
Transcriptomics combined with proteomics and echocardiography	miR-1, hs-cTnT, LV-GLS	Within 1 week after completion of anthracycline therapy in the induction phase of treatment	[45••]
	miRs-143-3p, miR-499a-5p, miR-142-3p, LVEF	Approximately 1 year after completing maximal therapy	[112]

### Proteomics combined with echocardiography

Due to their simplicity and reproducibility, proteomics and echocardiographic techniques, including sST2, MPO, cTn, NPs, and echocardiography, can be combined for effective clinical diagnoses. In particular, cTn, NPs, and other commonly used clinical biomarkers are often combined with imaging to diagnose CTR-CVT [96]. In a study by Sawaya et al. [97], hs-TnI combined with GLS (< 19% reduction) was significantly more sensitive (87%) for predicting trastuzumab-induced cardiotoxicity prediction compared with hs-TnI (74%) or GLS (48%) alone. In an observational study of radiotherapy patients, elevated sST2 levels were inversely correlated with GLS (*p* = 0.034) and LVEF (*p* = 0.006) levels at the 3-year follow-up [70]. In a multicenter cohort study, hs-cTnI and MPO, along with LVEF, were identified as predictors of cardiotoxicity risk in 78 breast cancer patients who received DOX and trastuzumab for 3 months. Additionally, the levels of three biomarkers MPO, PlGF, and GDF-15 were inversely correlated with LVEF over a follow-up period of up to 15 months (*p* = 0.02) [98]. Ky et al. [99] tested biomarkers such as CRP, MPO, GDF-15, PIGF, and sFlt-1 in breast cancer patients treated with anthracyclines and trastuzumab. They found that the combination of MPO and cTnI biomarkers at 3 months could assist LVEF in diagnosing subclinical cardiotoxicity. Lipschultz et al. [100] conducted a study on children with leukemia who were treated with anthracyclines for 4 years. The study found a positive correlation between the increase in cTnT during the first 3 months of chemotherapy and the thickness of the posterior wall of the left ventricular end-diastolic after 4 years (*p* < 0.01). Additionally, the study found that the increase in NT-proBNP was associated with an abnormal left ventricular thickness-to-size ratio (*p* = 0.01), indicating a higher risk of adverse left ventricular remodeling after four years; this suggested that NT-proBNP has additional predictive value when combined with echocardiography. Tan et al. [101] found that combining cTn with echocardiography, which includes GLS, led to a more precise prediction of CTR-CVT during the early stage of chemotherapy. The combination of NT-proBNP and LV-GLS is helpful in diagnosing mild left ventricular systolic dysfunction [102]. In a clinical observational cohort study examining NT-proBNP and echocardiography in 74 patients undergoing chemotherapy for breast cancer, there was a strong association between increased NT-proBNP levels and decreased LV-GLS in identifying cardiotoxicity. The results of the study showed good sensitivity and specificity [103].

### Metabolomics combined with proteomics and echocardiography

To achieve individualized precision medicine, multi-omics co-diagnostics can utilize metabolomics to accurately analyze changes in endogenous small-molecule metabolites [104]. This approach can help identify specific markers of CTR-CVT. Yuan et al. [105] conducted a study on a rat model of heart failure induced by anthracyclines and used a combination of metabolomics and proteomics to analyze rat tissues and plasma. The findings revealed that metabolic disorders, including those related to the tricarboxylic acid cycle, glutathione metabolism, glycolysis, glycerophospholipid metabolism, and fatty acid metabolism, contribute to the development of heart failure. Anthracyclines can affect aminoacyl-transfer RNA biosynthesis and interfere with glutamate, aspartic acid, and alanine metabolism by disrupting amino acid metabolism in the cardiotoxicity model [106]. In a clinical study, Asnani et al. [107] evaluated the role of intermediate metabolism in 38 breast cancer patients treated with anthracyclines and trastuzumab. Among the 71 metabolites measured, they found that changes in citric acid and aconitum acid predicted the risk of developing cardiotoxicity. In cardiotoxicity patients, the magnitudes of citric acid changes at 3 months were inversely correlated with changes in LVEF at nine months (*p* < 0.05). It is clear that plasma metabolites combined with echocardiography LVEF can effectively monitor the extent of myocardial damage.

### Cardiac magnetic resonance imaging combined with proteomics, transcriptomics, and echocardiography

The combination of CMR with proteomics and transcriptomics has promise in the personalized risk stratification of CTR-CVT. Clinical CMR results, when combined with biomarkers such as cTn and NPs, can enhance risk stratification [108] and enable observations of cardiac tissue structure. Harries et al. [109] conducted a study on 24 patients with hematologic malignancies who were undergoing anthracycline chemotherapy. The researchers conducted CMR and echocardiograms and sequenced circulating miRNAs at three different time points: before starting chemotherapy, upon completion of chemotherapy, and 6 months after completing chemotherapy. The results showed a positive correlation between CMR-derived mitral annular plane systolic shift (MAPSE) and LVEF (*p* < 0.05). Additionally, the expression levels of miRNA-5-221p and miRNA-3-2p were inversely correlated with LVEF (*p* < 0.05). CMR-derived MAPSE has been reported in several studies on hypertension and myocardial infarction as an independent factor for predicting cardiovascular outcomes [110]. In addition, MAPSE does not require sequencing and can be calculated quickly and easily from images. Therefore, combining CMR-derived MAPSE with LVEF for more frequent patient monitoring is achievable and has potential clinical value in predicting AIC and facilitating preventive treatment. In a large cohort study, Giusca et al. [111••] demonstrated that fast-SENC CMR, when combined with LVEF, cTnI, and BNP, can identify risk stratification in patients with heart failure. Furthermore, it can easily detect subclinical left ventricular dysfunction in some patients at risk of heart failure, even in the absence of structural and functional heart disease. This multi-omics model improves patient outcomes and minimizes adverse cardiac events by predicting risk, identifying high-risk individuals, and enabling individualized treatment options. It is more effective than relying solely on LVEF, cTnI, or BNP.

### Transcriptomics combined with proteomics and echocardiography

Combining transcriptomics with proteomics and echocardiography will facilitate the development of precision and individualized medicine in cardiac oncology. It will also assist patients in selecting cardioprotective strategies and alternative treatment options. Cheung et al. [50] conducted echocardiographic evaluations at all five time points when chemotherapy was administered in children with leukemia. They measured miR-1 and hs-cTnT levels and found that miR-1 significantly increased at the same time point when hs-cTnT levels peaked. Additionally, both miR-1 and hs-cTnT were negatively correlated with LV-GLS (*p* < 0.001). In this study, the plasma levels of hs-cTnT and miR-1 increased most significantly within 1 week after the completion of anthracycline therapy during the treatment-induction phase. Meanwhile, LV-GLS significantly decreased during this time period (*p* < 0.05), indicating severe cardiac muscle damage. Therefore, the study demonstrated that miR-1, hs-cTnT, and echocardiography can be combined to diagnose myocardial degeneration. Additionally, miR-1 combined with hs-cTnT may serve as a marker of myocardial injury during childhood leukemia treatment. In another study [112], it was found that children with malignancies were positively correlated with LVEF (*p* < 0.05) after approximately 1 year of anthracycline therapy alone. With continuous research advances in tumor cardiology, both in vivo and in vitro, the diagnostic model of transcriptomics combined with cTn and echocardiography has demonstrated significant potential in the early diagnosis of CTR-CVT. However, larger studies are required to validate these initial findings and to establish the role of miRNA combined with echocardiography in predicting cardiotoxicity.

## Discussion and prospects

Cardiotoxicity caused by anti-tumor therapy occurs when a drug’s action is not selective, i.e., when tumor cells are not specifically targeted. The harmful effects of multiple tumor treatment drugs on the heart may be additive or synergistic. Considering ongoing advancements and the development of new anti-tumor medications, CTR-CVT has emerged as a significant clinical concern. Therefore, it is crucial to effectively monitor cardiovascular adverse events resulting from anti-tumor therapy to ensure patients can safely and successfully complete their anti-tumor treatments. While certain clinical guidelines offer valuable guidance, there is a lack of consensus regarding effective management protocols. Cardio-oncology is an emerging and rapidly developing interdisciplinary field. Its purpose is to evaluate and manage the effectiveness of anticancer therapies and the risk of cardiotoxicity, providing a foundation for clinical decision-making [113]. The rapid development of precision medicine has provided us with a certain level of understanding of deep phenotyping. Integrated analysis of multi-omics can comprehensively explore biological characteristics and provide deep explanations of complex biological phenomena. This approach offers a new perspective for the diagnosis of CTR-CVT and overcomes the limitations of previous studies on single-omics diagnosis, enabling accurate disease diagnosis by leveraging the advantages of individual omics. Early warning, diagnosis, and monitoring of CTR-CVT based on multi-omics analysis technology have become the focus of research in recent years.

This article reviews the latest research and the limitations of single-omics techniques, such as genomics, transcriptomics, proteomics, metabolomics, and radiomics, in the diagnosis of CTR-CVT. It also describes the status, advantages, and prospective applications of joint multi-omics diagnosis. First, it is challenging to determine the optimal threshold and detection time and frequency for certain detection methods or biomarkers. It is also unclear whether detection thresholds for CTR-CVT differ from those of CVD alone, making it difficult to determine a multi-omics combination. Second, due to the complexity and heterogeneity of multi-omics big data, their reproducibility is difficult to ensure [114]; in addition, long-term studies are needed to determine the relationships between biomarker abnormalities and definitive clinical outcomes. In the field of cardio-oncology, there are many clinical reports, while basic research about the mechanism of CTR-CVT is lacking. Despite the widespread use of multi-omics techniques to discover potential biomarkers in recent years, a gap remains between research discoveries and their clinical applications. This gap can be attributed to the passive nature and design flaws of basic research, which results in low conversion to clinical practice. However, individual deep phenotyping and the utilization of big medical data are crucial components of precision medicine. It not only advocates for bench-to-bedside research but also requires two-way translational research from clinical treatment to community care. This includes moving from empirical medicine to evidence-based medicine and from micro research to macro industry. Precision medicine aims to discover and utilize heterogeneous treatment rules to inform reproducible, generalizable, and adaptable clinical decisions. Stratified medicine and tailored therapy require continuous effort to improve precision and personalization by incorporating new theories and data sources to enhance accuracy. How to facilitate two-way translational research, both from clinical to basic research and vice versa, is also an important issue.

With the increasing number of studies on omics, the challenge of improving and fully utilizing various research data has become a pressing issue. Fortunately, various techniques, such as artificial intelligence (AI) and machine-learning, have been proposed to analyze multi-omics data. These techniques aim to identify future directions for multi-omics features associated with disease phenotypes. By considering different types of molecular profiles, these techniques provide a comprehensive perspective and accelerate the discovery of candidate biomarkers for CTR-CVT screening and diagnosis. The characteristics of AI in automatic learning and intelligent decision-making determine its ability to process high-dimensional and complex data after CTR-CVT multi-omics analyses [115]. Therefore, AI is widely used in cardio-oncology image diagnosis and assists in decision-making, predictive early warning, individualized treatment, and other aspects. The application of AI technology in image analysis and diagnosis, such as echocardiography, electronic computed tomography (CT), ECG, and CMR, greatly improves the accuracy of cancer patient screening and follow-up [116–118]. It also quantifies cardiovascular risk and facilitates early identification of cancer patients at risk of cardiovascular complications [119]. For example, a random forest model including multiple variables was used to predict the occurrence of heart failure in patients with cancer-treatment-related cardiac dysfunction and reduced ejection fractions during a 3-year follow-up period [120]. These studies demonstrate the significant potential of AI in early warning and diagnosis of CTR-CVT.

## Conclusion

In the future, personalized medicine will emerge as the primary model for evidence-based and risk-specific precision medicine in cardio-oncology. Comprehensive risk assessment of CTR-CVT combined with traditional circulating biomarkers and new exploratory tools will pave the way for personalized cardiac safety predictions. This approach will also guide the management of CVD risk in future clinical practice in cardio-oncology.

## Data Availability

No datasets were generated or analysed during the current study.
